# Multimorbidity among inflammatory bowel disease patients in a tertiary care center: a retrospective study

**DOI:** 10.1186/s12876-022-02578-2

**Published:** 2022-11-26

**Authors:** Mahmoud H. Mosli, Majid Alsahafi, Mohammad N Alsanea, Faisal Alhasani, Mohammad Ahmed, Omar Saadah

**Affiliations:** 1 Department of Medicine, Faculty of Medicine, King Abdulaziz University, Jeddah, Saudi Arabia; 2 Inflammatory Bowel Disease Unit, King Abdulaziz University Hospital, Jeddah, Kingdom of Saudi Arabia; 3 Department of Pediatrics, Faculty of Medicine, King Abdulaziz University, Jeddah, Saudi Arabia

**Keywords:** Multimorbidity, Inflammatory bowel disease, Outcomes

## Abstract

**Background and objectives:**

Inflammatory bowel disease (IBD) is a chronic systemic inflammatory condition that debilitate the quality of life. Multimorbidity, a concept only beginning to emerge in IBD, is defined as two or more comorbidities present in the same individual. Notably, we used the term multimorbidity to refer to two or more comorbidities excluding IBD. Multimorbidity is linked to decreased quality of life, poorer disease outcomes, increased hospitalizations, healthcare costs and polypharmacy complications. We aim to estimate the prevalence of multimorbidity and to explore its effect on IBD patients.

**Methods:**

We retrospectively reviewed all IBD patients registered in a validated web-based registry since February 2018. Data on patient demographics, comorbidities, IBD and extraintestinal complications were obtained. We analyzed the date using univariate, bivariate and multivariable analysis.

**Results:**

Among 767 IBD patients, 54.6% had Crohn’s disease (CD), 41.9% had ulcerative colitis (UC) and 3.5% had IBD unclassified. The median age at diagnosis was 22 years (IQR: 15–29). Males compromised 50.2% of patients. According to the Montréal IBD classification, most UC patients had moderate UC (47.8%) while most CD patients had non-stricturing non-penetrating CD (49.8%). Overall, 10.3% IBD patients had multimorbidity and 23.9% had at least one comorbidity. The most common comorbidity was diabetes mellitus (4.9%) followed by essential hypertension (4%) and iron deficiency anemia (3%). Female gender (P = 0.008) and UC (*P* = 0.005) were more likely to have multimorbidity. Multimorbid IBD patients were more likely to develop thrombosis than non-multimorbid peers (16.7% vs. 1.6%; *P* < 0.001). Higher age at diagnosis (OR = 1.04, 95%CI: 1.01–1.07) and having a history of thrombosis (OR = 7.82, 95% CI: 2.67–22.92) are associated with increased risk of multimorbidity.

**Conclusion:**

Multimorbidity is not uncommon among IBD patients, especially females diagnosed with UC. Our findings indicate that future studies are needed to explore the effects of multimorbidity on IBD patients.

## Introduction

Chronic illnesses are still one of the most significant public health issues faced by populations worldwide [1]. Inflammatory bowel disease (IBD) is a chronic systemic inflammatory condition that is increasing in incidence globally [2, 3]. IBD can be recognized as an umbrella term that covers two lifelong diseases: ulcerative colitis (UC) and Crohn’s disease (CD) [4]. Both diseases are immune-mediated disorders characterized by relapsing and remitting inflammation of the gastrointestinal tract [5]. Despite the progression in understanding IBD, the exact etiology is still poorly understood [4].

IBD can significantly influence the quality of life and is linked to several multimorbidities such as colon cancer, ischemic heart disease, and cerebrovascular disease [6, 7]. In addition, multimorbidity is associated with decreased quality of life and level of function, as well as increased hospitalization, healthcare costs, demand for resources, physiological distress, complications due to polypharmacy, and mortality [8–10]. Multimorbidity is defined as the coexistence of two or more chronic diseases in one patient and this concept is only beginning to emerge in IBD patients [2, 11]. One study concluded that a multidisciplinary approach, with close monitoring of disease activity and early detection of comorbidities can benefit patients with IBD leading to better disease outcomes, improved response and adherence to treatment, quality of life, and decreased costs [2]. Another study conducted on IBD patients revealed that 78% of the patients had a minimum of 1 comorbidity with a median of 3 comorbidities [12].

These unforeseen numbers, in addition to the global burden, considerable healthcare costs, and the lack of studies confronting this topic in the literature, especially in the Middle East, demand more effort placed into the conduction of more research. Therefore, we aim to estimate the prevalence of multimorbidity and find the most common comorbidities among IBD patients. Additionally, we aim to determine the association between multimorbidity and IBD patient characteristics and care outcomes in a tertiary care center in Jeddah, Kingdom of Saudi Arabia.

## Materials and methods

After obtaining the ethical approval (Reference number: 433 − 21) from the institutional review board of King Abdulaziz University Hospital (KAUH), we conducted a retrospective study that included all patients diagnosed with IBD and registered in the IBD registry system of the IBD unit at KAUH. Since February 2018, the inflammatory bowel disease information system (IBDIS), a validated web-based registry system, has been used at KAUH to register, collect and store clinical information of all IBD patients who visited the outpatient clinic at KAUH. The data was retrieved on September 2021 which included all IBD patients registered in the IBDIS since its implementation at KAUH. No sampling technique was required since we included all IBD patients who were registered IBDIS. Variables including age, gender, type of comorbidity, number of comorbidities and smoking status as well as data on IBD such as type, severity, extent, course, intestinal and extraintestinal manifestations were obtained.

### Outcomes

The primary outcome of the study was to estimate the prevalence of multimorbidity, defined as the coexistence of two or more chronic diseases in one patient, among IBD patients. The main secondary outcome was to explore the impact of multimorbidity on disease outcome.

### Statistical analysis

Data was imported into Microsoft Excel 2016 and Statistical Package for Social Sciences (SPSS©) version 21 was used for data analysis. Frequency counts were used to summarize categorical variables while means with standard deviations (SD) or medians with inter-quartile ranges (IQR), depending on the distribution, were used for continuous variables. For bivariate analysis, the Chi-square test was used to compare frequencies, while the student t-test was used to compare means and the Mann-Whitney U test was used to compare medians. Multiple binary logistic regression analysis was used to determine the adjusted effects of various study variables on multimorbidity. For all statistical tests, a *p*-value of < 0.05 was defined as significant.

## Results

### Baseline characteristics

Our study population, as shown in Table 1, included a total of 767 IBD patients who were approximately equivalent in gender and predominantly diagnosed with CD (54.6%) with a median age of 24 years (17–33) at diagnosis. The majority of IBD patients were Arabs (95%) followed by Asians and Africans who were living in an urban environment (97%). Most of the patients did not have a history of cigarette smoking (86.2%).

**Table 1:** Descriptive characteristics of the patients, medians (IQR) or N(%)

N	Ulcerative Colitis	Crohn’s Disease	Unspecified IBD	Total
	321 (41.9%)	419 (54.6%)	27 (3.5%)	767 (100%)
Female	179 (23.3%)	187 (24.4%)	16 (2.1%)	382 (49.8%)
Male	142 (18.5%)	232 (30.2%)	11 (1.4%)	385 (50.2%)
Age at Diagnosis (median, IQR)	27 (19-39)	22 (15-29)	32 (20-50)	24 (17-33)
Ethnicity				
Arab	303 (39.8%)	396 (52.0%)	25 (3.3%)	724 (95.0%)
Asian	12 (1.6%)	10 (1.3%)	2 (0.3%)	24 (3.1%)
African	4 (0.5%)	10 (1.3%)	0 (0.0%)	14 (1.8%)
Smoking				
Ex-smoker	5 (0.9%)	11 (1.9%)	0 (0.0%)	16 (2.8%)
Current smoker	16 (2.8%)	45 (8.0%)	1 (0.2%)	62 (11.0%)
Non-smoker	207 (36.6%)	272 (48.1%)	9 (1.6%)	488 (86.2)
Environment				
Urban	288 (41.0%)	371 (52.8%)	23 (3.3%)	682 (97.0%)
Rural	7 (1.0%)	14 (2.0%)	0 (0.0%)	21 (3.0%)
Montreal Classification	Extent (E1) Ulcerative proctitis 62 (19.7%) (E2) Left-sided 126 (40.1%) (E3) Extensive 126 (40.1%) Severity Clinical remission (S0) 31 (9.9%) Mild (S1) 49 (15.7%) Moderate (S2) 150 (47.9%) Severe (S3) 83 (26.5%)	Location (L1) Ileal 114 (28.1%) (L2) Colonic 69 (17%) (L3) Ileocolonic 223 (54.9%) Behavior (B1) non-stricturing non-penetrating 205 (49.8%) (B2) Stricturing 119 (28.9%) (B3) Penetrating 88 (21.4%)		

Further analysis of our patients utilizing the Montreal IBD classification, 9.9% were in clinical remission (S0), 15.7% had mild UC (S1), 47.9% had moderate UC (S2), and 26.5% had severe UC (S3). As for the extent of UC, 19.7% had ulcerative proctitis (E1), 40.1% had left-sided UC (E2) and 40.1% had extensive UC (E3). On the other hand, CD patients had 28.1% terminal ileum (L1) involvement, 17% colonic (L2) involvement and 54.9% ileocolonic (L3) involvement. The behavior of CD in our sample showed that 49.8% had non-stricturing non-penetrating CD (B1), 28.9% had stricturing CD (B2), and 21.4% had penetrating (B3) CD behavior. As for the extra-intestinal complications of IBD, we summarized them in Table 2.

**Table 2:** Frequency and valid percent of IBD extraintestinal manifestations

Extraintestinal manifestation	N (Valid %)
Peripheral joint complications	195 (32.6)
Arthralgia	192 (32.1)
Mixture of type 1 and type 2 arthritis^a^	2 (0.3)
Type 1 Arthritis^a^	1 (0.2)
Stomatitis	82 (14.7)
Skin lesions	59 (7.7)
Erythema Nodosum	35 (4.6)
Pyoderma Gangrenosum	19 (2.5)
Psoriasis	5 (0.7)
Eye complications	41 (7.5)
Primary Sclerosing Cholangitis	25 (4.7)
Thrombosis	18 (3.3)

^a^Type 1 arthritis is mainly acute and remitting while type 2 is chronic with intermittent more severe flares

### Study outcomes

As shown in Table 3, we found that 184 patients out of the total IBD population had at least one comorbidity (23.99%); however, multimorbidity was identified in 79 (10.3%). Notably, we used the term multimorbidity to refer to two or more comorbidities excluding IBD. In addition, we found that diabetes mellitus (DM) is the most common comorbidity among IBD patients who had no other comorbidities (4.9%) and among patients who had multimorbidity (32.9%), followed by essential hypertension, iron deficiency anemia, hypothyroidism, and short stature due to an endocrine disorder [Table 4].

**Table 3:** Frequency of the number of comorbidities among all IBD patients

Number of comorbidities	N (%)
One comorbidity	105 (13.6)
Two comorbidities	46 (6.0)
Three comorbidities	23 (3.0)
Four comorbidities	7 (0.9)
Five comorbidities	3 (0.3)
Multimorbidity (≥2 excluding IBD)	79 (10.3)

**Table 4:** Frequency of the ten most common comorbidities

		All IBD patients	IBD patients with multimorbidity	IBD patients without multimorbidity	P*
**Rank**	**Comorbidity**	**N (%)**			
1	Diabetes mellitus	38 (5)	26 (32.9)	12 (1.7)	<0.001
2	Essential Hypertension	31 (4)	19 (24.1)	12 (1.7)	<0.001
3	Iron Deficiency Anemia	26 (3.4)	14 (17.7)	12 (1.7)	<0.001
4	Hypothyroidism	16 (2.1)	12 (15.2)	4 (0.6)	<0.001
5	Short stature due to endocrine disorder	11 (1.4)	7 (8.9)	4 (0.6)	<0.001
6	Vitamin D deficiency	9 (1.2)	5 (6.3)	4 (0.6)	0.001
7	Colorectal and anal malignant neoplasms	8 (1.0)	4 (5.1)	4 (0.6)	0.120
8	Osteogenesis Imperfecta	6 (0.8)	4 (5.1)	2 (0.3)	0.001
9	Diaphragmatic Hernia	4 (0.5)	3 (3.8)	1 (0.1)	0.004
10	Viral Hepatitis	2 (0.3)	2 (2.5)	0 (0)	<0.001

*P-values, assigning the deferences between multimorbid IBD and non-multimorbid IBD patients using Chi-square test.

### Bivariate analysis

Upon analyzing the relationships between multimorbidity and other variables as shown in Table 5, we found a significant relationship between multimorbidity and type of IBD (*P* = 0.009), gender (*P* = 0.008), age at diagnosis (*P* < 0.001) and thrombosis (*P* < 0.001). The majority of IBD patients with multimorbidity had UC (46.8%) while most patients without multimorbidity had CD (55.8%). Additionally, UC patients were more likely to have multimorbidity than CD patients (46.8% vs. 44.3%; *P* = 0.009). We also noticed that IBD patients with multimorbidity had a higher percentage of IBD-U diagnosis than those without multimorbidity (8.9% vs. 2.9%; *P* = 0.009). When it comes to gender, female patients were more likely to have multimorbidity than male patients (64.6% vs. 35.4%; *P* = 0.008). Notably, multimorbid patients were more prone to develop thrombosis than non-multimorbid patients (16.7% vs. 1.6%; *P* < 0.001). However, multimorbidity showed no significant statistical relationship with IBD relapse (*P* = 0.702), UC severity (*P* = 0.383) and smoking status (*P* = 0.810).

**Table 5:** The relationship of multimorbidity with different variables based on bivariate analysis

	Multimorbidity			
	Yes	No	Total	***p***
Type of IBD				
lcerative Colitis	37 (46.8)	284 (41.3)	321 (41.9)	0.009^1^
Crohn's Disease	35 (44.3)	384 (55.8)	419 (54.6)	
Unspecified IBD	7 (8.9)	20 (2.9)	27 (3.5)	
Gender				
Male	28 (35.4)	357 (51.9)	385 (50.2)	0.008^1^
Female	51 (64.6)	331 (48.1)	382 (49.8)	
Age at Diagnosis				
Mean (SD)	35.6 (18.3)	24.7 (12.3)	25.8 (13.4)	<0.001^2^
Thrombosis				
Yes	10 (16.7)	8 (1.6)	18 (3.3)	<0.001^1^
No	50 (83.3)	480 (98.4)	530 (96.7)	
IBD Relapse				
Infrequent relapse	44 (74.6)	384 (73.8)	428 (73.9)	0.702^1^
Frequent relapse	13 (22.0)	105 (20.2)	118 (20.4)	
No remission throughout a year	2 (3.4)	31 (6.0)	33 (5.7)	
Ulcerative colitis severity				
UC in clinical remission (S0)	2 (5.4)	29 (10.5)	31 (9.9)	0.383^1^
Mild UC (S1)	8 (21.6)	41 (14.9)	49 (15.7)	
Moderate UC (S2)	20 (54.1)	130 (47.1)	150 (47.9)	
Severe UC (S3)	7 (18.9)	76 (27.5)	83 (26.5)	
Smoking status				
Ex-smoker	2 (2.9)	14 (2.8)	16 (2.8)	0.810^1^
Smoker	9 (13.2)	53 (10.6)	62 (11.0)	
Non-smoker	57 (83.8)	431 (86.5)	488 (86.2)	

^1^ Chi-Square test ^2^ Independent samples t-test

### Multivariable analysis

The logistic regression model was evaluated based on an alpha of 0.05. The overall model was significant, χ2[9] = 34.8, *p* < 0.001, suggesting that age, gender, smoking status, type of IBD, IBD relapse and thrombosis had a significant effect on the odds of observing the multimorbidity. The regression coefficient for age at diagnosis was significant, OR = 1.0426, p = 0.001, indicating that age has a positive significant effect with multimorbidity. Among multimorbid IBD patients, more frequent cases of thrombosis were observed compared to non-multimorbid IBD patients (16.7% vs. 1.6%; Table 5, OR = 7.82 (95%CI: 2.67–22.92); Table 6). The rest of the variables in the model like smoking, gender, type of IBD and IBD relapse did not show significant effect on diagnosis. Details are summarized in Table 6.

**Table 6:** The relationship of multimorbidity with different variables based on logistic regression analysis

Predictor	Odds Ratio	95% CI		***p***
	Estimate	Lower	Upper	
**Age at diagnosis**	1.04	1.0162	1.07	0.001
**Smoking status**				
Ex-smoker – Non-smoker	0.74	0.0831	6.65	0.791
Smoker – Non-smoker	1.54	0.5431	4.37	0.417
**Gender**				
Male – Female	0.5	0.2353	1.05	0.067
**Type of IBD**				
Ulcerative Colitis – Crohn's Disease	0.96	0.48	1.91	0.910
Unspecified IBD – Crohn's Disease	2.36	0.03	199.14	0.704
**IBD relapse**				
Infrequent relapse – No remission	1.38	0.2801	6.75	0.695
Frequent relapse – No remission	1.24	0.230	6.68	0.801
**Thrombosis**				
Yes – No	7.83	2.6712	22.92	<0.001

Estimates represent the log odds of "Multimorbidity = Yes" vs. "Multimorbidity = No". Model Significance: χ2(9) = 34.8, *p* <0.001

## Discussion

In this study, the prevalence of IBD patients with multimorbidity, defined as two or more comorbidities in the same individual, excluding IBD was 10.3%. We also showed that 23.9% of IBD patients had at least one comorbidity. Patients with UC were more likely to have multimorbidity than patients with CD (46.8% vs. 44.3%; P = 0.009). However, this relationship could not be observed when adjusted for age, gender, smoking status, type of IBD, IBD relapse and thrombosis (OR = 0.96, 95%CI: 0.48–1.91). Although published research on comorbidities among IBD patients is limited, our findings agree with a previous Finnish study by Johanna et al. [13] reporting a percentage of 29% of IBD patients with at least one comorbidity. A further Swiss study by Carolin et al. [12] reported a percentage of 78% of IBD patients with at least one comorbidity, and a median of three comorbidities. The latter study also concluded that IBD patients have a higher prevalence of comorbidities compared to non-IBD patients. We believe that the variation in the proportions of IBD patients with comorbidities in the literature is due to the differences in the average age of IBD patients in each study as older IBD patients are generally more likely to have other age-related comorbidities.

Characterization of the comorbidities among patients with IBD is clinically relevant. We found that DM was the most common comorbidity among IBD patients (4.9%). In contrast, the Swiss study [12] found that DM was the eighth most common comorbidity among IBD patients with cardiovascular disorders being the most common comorbidity. The Finnish study [13] ranked DM fifth and chronic hypertension first in the comorbidities list among IBD patients. The different rankings of comorbidities among IBD patients can be explained by the natural epidemiological difference of diseases in different populations around the world. Moreover, the ranking of DM as the most common comorbidity in our study could point towards the high prevalence of DM in the general population of Saudi Arabia [14, 15]. However, this finding may be influenced by the use of diabetogenic medications such as corticosteroids.

One of the significant statistical relationships we found in our sample is the relationship of multimorbidity and the occurrence of thrombosis (P < 0.001). This finding was also supported by several studies [16, 17] which have shown an increased risk of venous thromboembolism (VTE) in patients with IBD. One cohort study showed a higher 3-year incidence of recurrent thrombosis in multimorbid patients [18]. However, dissimilar to our sample, the previously mentioned cohort study included acute VTE patients aged ≥ 65 years. Interestingly, our sample also showed a statical significance despite it not being formed of mainly old age patients with previous VTE. Since multimorbidity in IBD patients and thrombosis were statistically associated, physician should also have low threshold for suspecting thrombotic events in the multimorbid IBD population. However, this statistical association may be influenced by the type of medication which could alter the development of thrombosis. Nontheless, we believe that developing a specialized criteria for multimorbidity in IBD patients would be a beneficial tool to assess the risk of thrombosis and enhance the overall management of IBD patients.

The prevalence of multimorbidity in IBD patients is considerably worrisome as concomitant comorbidities can strongly influence the quality of life negatively in IBD patients [12]. Physicians treating IBD patients should be aware that appropriate management of comorbidities in IBD patients can lead to better IBD management outcomes and enhanced holistic patient-centered care. Furthermore, identifying certain comorbidities could significantly impact treatment selection, specifically by avoiding medications that predispose to infections or even thrombosis. For example, patients who are older than 55 and have DM might be better served by treatment with a leukocyte trafficking inhibitor such as vedolizumab rather than an anti-tumor necrosis factor agent (anti-TNF), which have been associated with higher risk of serious infections [19]. A recent recommendation by the European Crohn’s and Colitis Organization (ECCO) suggested that the use of Janus kinase inhibitor tofacitinib should be avoided in elderly patients who have multiple cardiovascular risk factors as data from open label studies conducted in rheumatoid arthritis suggest a higher risk of venous thromboembolism in this patient cohort [20].

There is also increasing evidence to support a plausible association between IBD and several comorbidities, related to poor disease outcomes, higher mortality, and increased hospitalization rates [2, 12]. We suggest further research on the prevalence of multimorbidity among IBD patients on wider scales in terms of both sample size and demographics, and we also urge researchers to further investigate the effects of multimorbidity and polypharmacy on IBD patients. Major strengths of the present study include the limited published research on the topic of multimorbidity among IBD patients and the reliable comprehensive data set available for analyses. Nevertheless, a major limitation of our study is the usual challenge in retrospective studies to obtain accurate conclusive patient outcomes and to determine the precise timeline of events after the diagnosis. Another major limitation is the absence of some variables such as the type and number of IBD medications and non-IBD medications used in different time points which would have been beneficial in assessing the impact of polypharmacy on IBD course, management and patient care outcomes.

In conclusion, our findings demonstrate that multimorbidity is not uncommon among IBD patients, especially females diagnosed with UC. Thrombosis and multimorbidity in IBD were statistically associated; therefore, scales for the assessment of thrombosis might be beneficial for improving the overall management of multimorbid IBD patients. Our study also highlights the importance of recognizing common comorbidities in IBD patients to develop a multidisciplinary management approach. Future studies evaluating the impact of multimorbidity on IBD course, management and patient care outcomes are needed to enhance the holistic care of patients.

## Data Availability

The datasets used and/or analysed during the current study are available from the corresponding author on reasonable request.

## Abbreviations

IBD: Inflammatory bowel disease
UC: Ulcerative colitis
CD: Crohn’s disease
KAUH: King Abdulaziz University Hospital
IBDIS: Inflammatory bowel disease information system
SD: Standard deviations
IQR: Inter-quartile ranges
DM: Diabetes mellitus
VTE: Venous thromboembolism
TNF: Tumor necrosis factor
ECCO: European Crohn’s and Colitis Organization
